# Rapid Assessment Response (RAR) study: drug use, health and systemic risks—Emthonjeni Correctional Centre, Pretoria, South Africa

**DOI:** 10.1186/1477-7517-11-11

**Published:** 2014-04-03

**Authors:** Monika ML dos Santos, Franz Trautmann, Gustaaf Wolvaardt, Romeo Palakatsela

**Affiliations:** 1 Psychology Department, University of South Africa, P.O. Box 392, UNISA, Pretoria 0004, South Africa; 2 International Affairs, P.O. Box 725, NL-3521 VS, Utrecht Trimbos Institute 3500, The Netherlands; 3 Foundation for Professional Development, P.O. Box 75324, Lynnwood Ridge, Pretoria 0040, South Africa

**Keywords:** Correctional Services, Drug use, Infectious diseases, Health promotion, Harm reduction, South Africa

## Abstract

**Background:**

Correctional centre populations are one of the populations most at risk of contracting HIV infection for many reasons, such as unprotected sex, violence, rape and tattooing with contaminated equipment. Specific data on drug users in correctional centres is not available for the majority of countries, including South Africa. The study aimed to identify the attitudes and knowledge of key informant (KI) offender and correctional centre staff regarding drug use, health and systemic-related problems so as to facilitate the long-term planning of activities in the field of drug-use prevention and systems strengthening in correctional centres, including suggestions for the development of appropriate intervention and rehabilitation programmes.

**Method:**

A Rapid Assessment Response (RAR) methodology was adopted which included observation, mapping of service providers (SP), KI interviews (staff and offenders) and focus groups (FGs). The study was implemented in Emthonjeni Youth Correctional Centre, Pretoria, South Africa. Fifteen KI staff participants were interviewed and 45 KI offenders.

**Results:**

Drug use is fairly prevalent in the centre, with tobacco most commonly smoked, followed by cannabis and heroin. The banning of tobacco has also led to black-market features such as transactional sex, violence, gangsterism and smuggling in order to obtain mainly prohibited tobacco products, as well as illicit substances.

**Conclusion:**

HIV, health and systemic-related risk reduction within the Correctional Service sector needs to focus on measures such as improvement of staff capacity and security measures, deregulation of tobacco products and the development and implementation of comprehensive health promotion programmes.

## Background

Of all regions in the world, sub-Saharan Africa is the hardest hit by the HIV epidemic, with almost two thirds of all people infected living in the region [1]. Correctional Service populations are one of the populations most at risk regarding HIV infection for many reasons including unsafe injecting practices, unprotected sex, violence, rape and tattooing with contaminated equipment [2]. Despite this, and although there has been a significant increase in national and international funding to control the epidemic, correctional centre settings in sub-Saharan Africa have received surprisingly little attention, and limited studies have been conducted in South Africa that explore the links between drug use, health and systematic risks for both offenders and staff.

The information that is available on the situation in South African correctional centres indicates that living, health and social conditions of offenders are worrisome. Correctional centres are overcrowded, living conditions are poor and insufficient health services are evident [3]. Reports of gangster rule and widespread (sexual) violence as well as drug availability are prevalent, and links with the community are minimal, resulting in lessened prospects for rehabilitation [4]. The data and information available underline this picture as can be taken from the following examples: South Africa has an extraordinarily high rate of incarceration (413 per 100,000) compared to other low-income and middle-income countries in Africa (for example, Mozambique 50, Tanzania 116 and Libya 207) and other parts of the world (for example, India 29, China 118 and Iran 191). Only Russia and Belarus (532), Cuba (±487) and Ukraine (416) score higher [5]. The Department of Correctional Services HIV Prevalence Survey undertaken nationally in 2006 indicates that 19.8% of offenders tested positive for HIV in 2006, the majority (81%) falling in the age group 26–45 [6]. The availability of drugs of abuse through corrupt staff and centre gangs within the Correctional Service system in South Africa is concerning [4]. A cross-sectional study conducted in 2007 amongst 357 offenders across four South African correctional centres involved in a pre-release intervention programme for parolees indicates that offenders demonstrate high levels of substance use and engagement in risky sexual behaviours. Furthermore, outcomes of the same study suggest that never-using condoms before was highly associated with lower intention to engage in preventive behaviours upon release [7]. Overall, specific data on drug users in correctional centres is not available for South Africa, as well as the majority of countries. The threat of tuberculosis (TB) and drug-resistant TB are all more crucial concerns, given the inferred high HIV prevalence in southern African correctional centres, and in the general population. HIV prevalence in the region's correctional centres has been estimated to be between 2 and 50 times than that outside correctional centres [8]. This figure also applies worldwide [9]. Higher levels of sexual risk behaviour—including sexual violence—also prove to be more common among young males in correctional centres when compared to the community [3,10].

### Study aim

The study aimed to identify the attitudes and knowledge of key informant (KI) offender and correctional centre staff regarding drug use (both illicit drugs and those drugs used illicitly within the centre) and the mapping of drug use, health and systems-related problems so as to facilitate the long-term planning of activities in the field of drug-use prevention and systems strengthening in correctional centres, including suggestions for the development of appropriate interventions.

### Study objectives

Specific objectives were:

• To obtain a reliable impression of the nature and extent of drug use, health and systems-related problems, such as HIV, among offenders in one selected correctional centre.

• To assess the (priority) needs of offenders and staff with regard to relevant health and social care interventions related to drug use.

• To identify the required and feasible responses (adapted to the South African context) so as to effectively tackle identified drug use, health and systemic problems.

• To analyse and describe the conditions in the selected correctional centre, taking into account the South African correctional service system, when realising and implementing these responses.

## Methods

The core activity encompassed a qualitative assessment using the Rapid Assessment Response (RAR) methodology, an approach which is practice- and need-driven, linking directly the identification and planning of appropriate responses to the outcomes of the assessment [11]. The study included observation, mapping of related service providers (SP) (in order to help existing services develop their practices further if needed), KI interviews (15 staff and 45 offenders) and focus groups (FGs) with offender participants (*n* = 36) and staff (*n* = 4). The assessment tool is based on the Rapid Assessment and Response Guide on Injecting Drug Use (IDU-RAR) [12]. The RAR methodology was initially developed by the Centre for Research on Drugs and Health Behaviour at the University of London for WHO and UNAIDS and has been tested in various projects in the field of drugs and addiction—and is particularly suitable for investing problems within public health without resorting to ‘unscientific’ speculation—and which at the same time provides instruments and data for concrete intervention planning [13-16].

The focus of RAR is on relevance to intervention and pragmatism, rather than scientific perfection. For adequate interventions in the field of health promotion, there is no need to know the absolute number of people involved in a certain risk behaviour. It is sufficient to have cognisance that a substantial number of people are involved in this risk behaviour [15].

### Ethical approval, participants and setting

Ethical approval for the study was obtained from the South African Department of Correctional Services Research Ethics Committee (DCSREC) in June 2011, and consent to disseminate study findings were obtained from the South African Department of Correctional Services in July 2013. The study was implemented in Emthonjeni Youth Correctional Centre, Baviaanspoort Correctional Centre, Pretoria, South Africa. The Baviaanspoort management area comprises four centres, namely, maximum (sentenced adults, approximately 600 offenders), medium (sentenced male adults, approximately 1,200 offenders), Emthonjeni (sentenced youth, between 320 and 380 offenders) and community corrections (parolees). Key selection criterion for KIs from correctional centre management and staff was commitment to the idea of improved health and social situations in the centre. They also needed to display a genuine interest in developing and implementing drug use, health, systems and social interventions and programmes as presented in the recommendations of the assessment undertaken in this project. KI offender participation selection criteria were based on those who felt that they could provide a meaningful contribution to the study and who displayed a willingness to participate in a study of this nature. Correctional Service staff, together with research staff, introduced the research study to the offenders, and those offenders prepared to participate were either individually interviewed by the research fieldworkers or as part of a focus group. The participants were not compensated for their participation in the study and were assured that all information that they provided would be kept confidential within the limitations of the law and that they had the right to refuse to answer any particular questions or to withdraw at any stage of the study without incurring any negative repercussions to themselves. The study aimed to include a total of 60 KI individual interview participants, 20 being KI correctional centre staff, and 40 KI offender participants. However, due to staff shortages and time constraints, only 15 KI staff participants were interviewed and 45 KI offender participants. The implementation of the study in Correctional Services spanned a 2-month period.

The researchers did not have access to the personal files of offender participants; however, the centre's offender population is male, between the ages of 14 and 35 (in South Africa, youth is classified as an individual between the ages of 14 and 35). The centre covers the spectrum of illegal crimes—ranging from murder, theft, housebreaking, rape, illegal drug possession and drug trafficking. Participation was undertaken anonymously with participant codes documented instead of names. All information was dealt with confidentially and will be kept in a secure room for a minimum period of three years after the finalisation and publication of study outcomes.

### Semi-structured interviews and focus groups

A semi-structured questionnaire was developed for individual interviews. To structure and organise the RAR, a set of key questions were developed, comprising central questions for collecting information about drug use (illicit drugs and those drugs prohibited by the correctional centre but still illicitly used by offenders), systemic risks and adequate responses. These formed the basis and framework for all phases of information collection. The following set of key questions was adopted:

• Who is using drugs in a problematic way within the correctional centre?

• What drugs are used in the correctional centre?

• How are drugs used in the correctional centre?

• What health risks are involved in drug use?

• What do staff/offenders know about these drug-related health risks?

• What do drug users do to avoid these health risks?

• What drug-use/HIV-related interventions/services are available within the correctional centre system?

• What drug-use/HIV-related interventions/services are realisable within the correctional centre system?

• What other health and systemic risks are encountered in the correctional centre?

The questionnaires were completed anonymously, and all KI participants provided informed consent prior to the commencing of the interview. All individual interviews with KIs were conducted by interviewers in the field of psychology.

The information collected through the questionnaires served as background information for the FGs with selected stakeholders and participants. Two FGs were conducted, the first with KI offenders, and the second with KI Correctional Service staff members. The FGs strove to verify collected information and to find explanations for diverging or contradicting information, and to reach consensus on appropriate and feasible interventions. Based on the findings from the interviews, the study investigators selected the following issues for the FGs:

• Clarification on questions regarding drug use in the correctional centre (different views on types of drugs used, etc.).

• Availability of drugs (is availability stable?).

• Are drugs of choice (the drug favoured by an offender at time of participating in the study) available in the correctional centre (in the interviews, staff tended to say no, while offenders tended to say yes)?

• What are the important health risks for staff (in the interview, there are differences in views: offenders said violence, staff said health risks).

• Frequency of violence in the correctional centre.

• What are important (drug use-related and other) health risks for offenders?

• Staff capacity (for example social workers, health staff, etc.).

• Training needs of staff.

• Information and health care needs of offenders.

• Activities for offenders.

Data was collected by means of the individual questionnaires that were conducted by trained fieldworkers studying in the field of psychology, and notes were taken by two of the study investigators and one of the field workers during the focus groups (with both the offenders and staff). All raw data from the questionnaires was captured on an Excel spread sheet, and data from the focus groups were categorised according to themes also on an Excel spread sheet.

### Data analysis

Thematic content analysis was adopted to analyse the interview information, such methods have been shown to be particularly valuable in the development of public healthcare interventions. Thematic coding was adopted by the investigators in the analysis for the disaggregation of core themes; it is a multi-step process during qualitative data analysis that encompasses a process of relating codes (categories and concepts) to each other, via a combination of inductive and deductive thinking. To authenticate interpretations, a draft research report and study conclusions were taken back to a subset of staff participants and other correctional staff for enrichment and verification of interpretations [17,18]. Unfortunately, it was not possible to obtain any additional input concerning study conclusions from offender participants.

## Results

### Offender and staff participant demographics

The majority of KI offender participants were aged 19–21 years old (32/45), with a range from 18 to 31 years old. Thirty-six of the KI offenders were from a black ethnicity. Grade 10 was the highest level of education for 18 of the KI offenders. Most KI offender participants were South African citizens (4/45), and two were Zimbabwean. The majority of KI staff participants were female (9/15). The age range of KI staff participants was from 28 to 48 years old (Table 1).

**Table 1:** Offender participant demographics

	**Number**	**Percentage**
Age		
< 18	3	7
19–21	32	71
22–24	8	18
25–27	1	2
28–30	0	0
31+	1	2
Ethnicity		
Black	35	78
Coloured	9	20
White	1	2
Citizenship		
South African	43	96
Zimbabwean	2	4
Length of drug use prior to incarceration		
Did not use	6	13
0–11 months	6	13
1–2 years	2	4
3–4 years	16	36
5–6 years	4	9
7+ years	4	9
Types of drug used before incarceration		
Alcohol	5	9
Cannabis	3	5
Heroin	3	5
Crack cocaine	3	5
Mandrax	3	5
Poly substances	38	69
Highest level of education		
Grade 1	1	2
Grade 2	0	0
Grade 3	1	2
Grade 4	1	2
Grade 5	2	4
Grade 6	1	2
Grade 7	0	0
Grade 8	6	13
Grade 9	7	16
Grade 10	18	40
Grade 11	2	4
Grade 12	3	7
College	1	2
Unknown	2	4
Prior incarceration		
Yes	19	42
No	26	58
How many years in prior incarceration (years)		
0	24	58
1	4	9
2	9	20
3	4	9
4	1	2
5+	1	2
Time spent in current sentence (years)		
< 1	21	47
1–2	19	42
3–4	5	11
5+	0	0
Remaining current sentence time (years)		
< 1	17	38
1–2	18	40
3–4	5	11
5+	2	4
7+	3	7

### Offender drug use

Sixteen of the KI offender participants had used a range of drugs for between 3 and 4 years before being incarcerated, while 13 had used drugs for between 1 and 2 years prior to being incarcerated. Thirty-eight KI offender participants stated that they used a combination of drugs (two or more) before entering the correctional centre; this ranged from a broad spectrum of substances such as inhalants combined with cannabis and alcohol to substances such as ecstasy.

### Views related to drug use and health in the correctional centre

#### *Types of drugs used before entering the correctional centre*

Heroin/cannabis mix (referred to as ‘nyaope’—a South African township culture term) was identified as the most common drug used before coming to the correctional centre (*45*/45), followed by crack cocaine (44/45) and multiple other drugs (inhalants, ecstasy, etc.) (40/45). The KI staff participants were of the opinion that nyaope is the most common drug used before entering the correctional centre (15/45). Other drugs cited include cannabis (13/15), cocaine (5/15) and tobacco (2/15).

#### *Number of offenders using drugs in the correctional centre*

Most KI offender participants (44/45) felt that the majority of offenders used drugs before entering the correctional centre. The majority of KI staff (10/15) were also of the opinion that offenders use drugs in the correctional centre.

#### *Number of offenders who started using drugs in the correctional centre*

A large proportion of KI offenders (27/45) were of the opinion that <10% of offenders in the centre started using drugs in the correctional centre. Similarly, the majority of KI staff participants (8/15) were of the opinion that <10% of offenders started using drugs in the correctional centre.

#### *Type of drugs used in the correctional centre, drug availability and changes in availability over the past 12 months*

The most commonly used drugs in the centre, according to the KI offenders, is cannabis (35/45) and nyaope (21/45). The KI staff participants also felt that cannabis (15/15) was the most commonly used drug in the centre, as well as best blend (BB) tobacco (4/15). Drug supply was generally thought to be low in the last 12 months by all KIs. The KIs were generally of the opinion that drugs of choice, defined as the favoured drug of an individual at a specific point in time, for the most part are not available in the centre (28/45) or not very easily available (4/45).

#### *Method of drug administration*

The most common method of drug administration cited by all KIs was smoking (40/45); the second most common mode of drug administration mentioned was injection at 17% (9/45). The KI staff participants also stated that smoking (15/15) was the most common mode of drug use in the correctional centre.

### Health risks for staff and offender population and knowledge of health risk

Eleven of the KI offender participants were of the opinion that the greatest risk for staff was being stabbed by an offender, followed by the risk of TB contraction (5/45). For KI offender health risk, HIV and AIDS contraction was mentioned by eight of the participants. They felt further that other great health risks for offenders included TB contraction (7/45) and being stabbed (5/45). KI offender participants thought that poor living conditions, for example, the quality of the plumbing and cells, contributed to health risks (21/45), as well as social situations such as non-consensual penal-anal penetration (20/45), socio-economic conditions (20/45) and geographic origin (10/45) (drug availability in city centres tends to be more prevalent than in rural areas for example) contributed to drug use. The KI staff participants felt that the most serious health risks for them were TB contraction (9/15), HIV contraction (8/15) and being assaulted by offenders (7/15). They further imparted that the most serious health risks for offenders included TB contraction (11/15), HIV contraction (11/15) and hygiene problems (9/15).

Some key informant offender participants felt that offenders are aware of TB (16/45) and HIV (12/45) drug-related risks. Most KI offender participants felt that staff have overall good knowledge about protecting against health risks (33/45) by wearing surgical gloves for example. There were mixed responses in terms of offender knowledge of protective measures against health risks; some protective measures that offenders use that were mentioned included not sharing needles (4/45), abstaining from sex and penal-anal penetration (3/45), using condoms (2/45), covering mouths when coughing (2/45), quitting drug use (3/45), eating fruit (1/45), using Salvon (1/45; Johnson & Johnson, New Brunswick, NJ, USA) and exercising (1/45). One offender mentioned protective measures that seemed inadequate, for example, crushing cannabis first. Some KI staff participants felt that there is a fair degree of knowledge regarding HIV contraction (6/15), TB contraction (6/15) and mental illness (6/15). The KI staff participants were of the opinion that offenders were not that well aware of protective measures (8/15), although they felt that some offenders do have knowledge about condom usage (4/15) and keeping windows open (4/15), for example, so as not to contract TB. KI staff participants felt that offenders primarily attend workshops (14/15) in order to learn what to do to protect themselves from drug-related risks.

### Offender focus group findings

Thirty-six offenders participated in the FGs. It resulted in the following findings:

#### *Drug use in the correctional centre*

Many offenders become incarcerated due to drug use-related problems, and they need to cope with their addiction within the correctional centre setting. Withdrawal symptoms are treated symptomatically within the system, and no pharmacotherapy maintenance regimes are offered. The KI offenders stated that heroin (mostly mixed with cannabis, or nyaope) is not that easy to obtain within the correctional centre system. However, according to the KI offender participants, drugs are smuggled into the correctional centre by officials, family and other visitors (these include substances such as tobacco, cannabis and sleeping pills—which offenders then smoke with BB tobacco, Lancaster, PA, USA). KI offender participants stated that if an offender has a close relationship with a centre official, he will be able to get his drug of choice. Offenders are also able to smuggle drugs from the adult medium division, which neighbours the juvenile correctional centre. The KI offenders did feel, however, that it is harder to access drugs in the correctional centre than in a rehabilitation centre.

#### *Health risks for staff and offenders*

Tuberculosis was cited as a health risk within the correctional centre cells as they are not well ventilated, and between two and four offenders sleep in a cell approximately the size of a small bathroom (3.192 m^2^). The KI offender participants stated that there is a high degree of peer pressure within the system and that consensual penal-anal penetration in particular (often transactional in nature) is an extensive problem. Transactional sex often occurs when offenders are hungry or looking for drugs and subsequently voluntarily exchanging sex for food or drugs. Condoms are available in the correctional centre, but not lubrication. Offenders stated that they do make use of condoms. HIV tests can be undertaken in the correctional centre hospital on a voluntary basis. Emotional and psychological issues are evident amongst offenders due to being raped, transactional penal-anal penetration and other traumatising experiences. KI offender participants stated that they come out of the correctional centre a different person. The KI offenders also stated that the lack of visits from their families and significant others increases the rate of penal-anal penetration due to emotional and psychological reasons.

#### *Staff capacity, management and activities for offenders*

KI offender participants felt that there was overall poor central management at the centre. KI offenders were of the opinion that they could recover from drug addictions but that they needed motivation and encouragement to do so. The programmes mainly available in the correctional centre include the following: anger management, courses for offenders whose convictions relate to sexual offences and HIV and AIDS programmes. They also expressed a wish to be able to choose for themselves which programme to attend. KI offender participants felt that educational workshops could improve, and they stated that some offenders do not know how to read and write. Drug rehabilitation is not readily available, and many offenders struggle with their drug addiction syndrome while they are in their cells. They further stated that boredom within the system poses an extensive problem.

### Staff focus group findings

Four KI staff members participated in the FGs: a social work manager, a teacher, a nurse and a unit manager.

#### *Drug use in the correctional centre*

The drugs mainly used in the correctional centre, according to the KI staff participants, are tobacco and cannabis. The KI staff further stated that offenders are not allowed to smoke cigarettes—this was a decision taken by Emthonjeni Correctional Centre Head and middle management and not across Correctional Services per se. A number of years back, they were allowed to smoke cigarettes, and these were sold on the premises. However, small quantities of cannabis and tobacco are flushed away, and offenders are not prosecuted if these substances are recovered from cells (as it requires a great deal of logistics to go to court). Offenders fight over BB tobacco (Lancaster, PA, USA) and other drugs and will also sell their food to obtain these substances. Some time ago, staff members were also caught using drugs; staff members are not searched when they report for duty.

#### *Health risks for staff and offenders*

Tuberculosis is not really a problem according to the staff KI participants—only two offenders had reported TB at the time this study was conducted. In 2011, 105 offenders were tested on a voluntary basis for HIV (out of approximately 380 offenders)—two were tested positive. The building quality of the correctional centre is also not good—and thus makes it more prone to vandalism. Certain parts of the centre are consequently not operational due to vandalism and have incurred damage such as leaking pipes. Due to this, there is overcrowding in the sections that are operational, leading to a greater risk for cross-infection. According to the staff participants, offenders are knowledgeable about HIV and AIDS risks. However, transactional sex is a regular phenomenon, and according to KI staff participants, if the transaction did not go according to plan, it often leads to rape. Gangsterism is a pervasive problem in the centre and has various negative health consequences. Generally, gang members acquire their ranks by doing something such as rape and stabbing, which can pose a serious health risk to both offenders and staff—although this normally takes place within the maximum correctional centre.

#### *Staff capacity and management*

A shortage of manpower and availability of staff is evident in the correctional centre. The KI staff participants emphasised the need for improved manpower in terms of security, nurses and social workers. The 12-h shifts/7-day week that security staff work is problematic because there is no work endorsement from one shift to the next. In reality, there are only two security staff members for 170 offenders as frequently about six staff members are out on offender escorts to court, hospital, etc. The standard ratio of security staff to offender is meant to be 1:40. Nurses render standby duties to the centre and other sections of Baviaanspoort Correctional Centre after 1600 hours—there are four nurses for the centre, and they get called out approximately three to four times a month. Currently, there is only one social worker for approximately 370 offenders (instead of the required three) who also serves as a manager, although she has no subordinates. She also has to compile all the offender parole reports. Although there are vacancies for another two social workers, they have not been filled for a substantial period of time. One psychologist runs programmes in the four centres in Baviaanspoort and has also other duties to fulfil, which implies that offenders are not receiving substantial psychological care. Pepper spray, shields and panic buttons (which are ineffective as the staff demonstrated them to the interviewers and there was no response from an emergency team) are made use of by the staff; however, they receive no training or demonstrations on how to make use of these equipment. Staff members are also not allowed to bring in their cell phones. Furthermore, officials do not carry guns in a correctional centre; there is an emergency team, which is only called in for a serious situation. All these factors contribute to the stress levels of staff; burnout and alcoholism are common problems amongst staff. Overall, the KI staff feel very unsafe, for example, there is one female official who locks up about 100 offenders alone in Block C (there are meant to be a minimum of six officials, but others are normally out on escorts or on leave). There is also no proper training on programmes which run in the correctional centre. Life skills programmes were stopped in 1999. Programmes were downloaded from the internet website, and social workers were simply requested to run the programme.

#### *Information, activities and health care needs of offenders*

The KI staff participants stated that most drug-using offenders are referred to a psychiatrist. The psychiatrists come to the facility based on the need for services. Heroin addiction is treated mainly with benzodiazepines and according to the offenders' withdrawal symptoms; no pharmacotherapy maintenance regimes are offered.

## Discussion

Four clusters of problems can be discerned which are worth further consideration:

### Drug use in the correctional centre (including tobacco)

Study findings indicate that a large proportion of offenders use drugs before entering the correctional centre, similar findings to that in the survey undertaken by Stöver and Michels [19]. Heroin and cannabis mixed together and smoked, which were most commonly used prior to incarceration, are also drugs that are prevalent within the greater vicinity of Tshwane [20]. Both KI offenders and staff state that drugs are smuggled into the centre by officials, by visitors and from the adult sections of the correctional centre. Notwithstanding, the KI offenders did feel that it is harder to obtain drugs in the correctional centre than in a rehabilitation centre—this may inadvertently provide an ideal window period for proactive rehabilitation programming. An encouraging outcome of the study is that offenders addicted to heroin, for example, are treated symptomatically for their withdrawal symptoms; however, there may still be a need for pharmacological maintenance regimes within correctional service facilities in South Africa. The study by Stöver and Michels [19] demonstrates how the implementation of opioid substitution therapy (OST) programmes, for example, benefits not only offenders but also correctional centre staff and the community at large, and reduces the rate of infectious diseases. There is one specific feature of Emthonjeni which is worth some consideration: past Emthonjeni Correctional Centre Head and middle management banned tobacco and cigarette smoking a number of years ago in an effort to protect the health of the youth offenders. Unfortunately, this measure proves to have some negative unintended consequences, in particular, bringing about and maintaining black-market features such as smuggling and transactional sex in order to obtain cigarettes and violence (fighting about tobacco deals), features comparable to the illicit underworld. Moreover, the tobacco ban is causing a great deal of unnecessary (monitoring) work by staff.

### Health risks of staff and offenders

According to the KI offenders, violence is the most important health risk for staff, while staff regarded infectious diseases as the major health risk for themselves. Overall, the frequency of (serious) violence against staff seems to us rather low. However, having discussed this with the KI offenders and staff, the impression is held that this is the result of luck rather than security policy. The measures taken to secure staff are, according to the study investigators, insufficient. Staff members have a panic button, a cell phone-sized device producing a danger signal which is relatively loud. Experimenting with it by some of the study investigators and KI staff showed that nobody responds to the alarm. There are no clear instructions or trainings for staff on how to deal with (threats of) violence. The development of a best practice comprehensive security plan explicitly describing measures and means is a necessity, for example, a device that includes GPS enabling the other staff/the control room to locate a staff member in danger.

The provision of condoms by the correctional services is in line with public health recommendations; however, the provision of condoms without lubricant is a public health issue due to increased risk of physical trauma. Regarding other health risks for offenders, the staff's emphasis on infectious diseases was evident. Offenders also recognise this problem, but their view is rather diverse, mentioning a variety of health risks. The risk for HIV contraction is also fuelled by a black market of illegal drugs and prohibited tobacco (and related transactional and coerced sex that occurs). Furthermore, offender participants stated that injection drug use (IDU) was the second most common mode of drug administration, and IDU is a known contributor to the spread of HIV infection. Further research is needed to corroborate this finding as staff do not mention it, and a review of the literature suggests that no studies have been undertaken in South Africa focusing on IDU in correctional settings. Surprisingly enough, the KI staff participants reported that in 2011, 105 (out of approximately 380) offenders were voluntarily tested for HIV, and only two tested HIV positive. It is uncertain if this is a generalisable finding to the rest of the offenders in the centre, or if offenders who have already been tested positive or suspect that they are HIV positive were reluctant to be tested; there may also be other unidentified reasons for possible underreporting of HIV, suggesting a need for further research on this aspect. There is a fair chance that both HIV and TB are underreported by offenders and therefore occur at a rate higher than that which is officially reported. Part of the problem is that the statistics regarding the number of HIV-positive offenders are unknown. The Department of Correctional Services estimates the figure to be 3% [6]. Yet, Goyer [21], a researcher with the Institute for Security Studies, currently places the figure at a little over 40%, while Inspecting Judge of Correctional Service Centres Johannes Fagan suggests that the figure could be as high as 60% [21]. There exists only one instance of independent research regarding HIV prevalence in South African correctional centres. The research was undertaken in Midlands Medical B, a correctional centre in the province of KwaZulu-Natal, an area with a very high HIV infection rate among the general population. The report has been withheld by the Department of Correctional Service due to fears that it might be used as an indicator of figures for the correctional centre system as a whole. This basic lack of knowledge is a key problem facing those looking to fight the problem. Whatever the actual rate of infection, it is undeniable that HIV and AIDS are having an impact on the Correctional Service population in South Africa. Goyer's [21] research revealed a 750% increase in the number of natural deaths in South African correctional centres since 1995. Of these, 90%–95% are attributed to diseases often associated with the weakened immune system that AIDS produces; Goyer's [21] figure of the role of AIDS in correctional centre deaths is further supported by a study of postmortem reports carried out in 1995 that placed the number of correctional centre deaths from similar diseases also at 90%. Additionally, Fagan's report showed a record number of natural deaths in correctional centres, with 1,169 in 2001, an increase over 600% from 186 in 1995. The South African justice system releases and takes on approximately 25,000 offenders every month [21]. This emphasises the fact that the problem of HIV and AIDS in South African correctional centres cannot be seen as an isolated problem, but rather as one that has a serious effect on South African society as a whole [22]. Furthermore, in correctional centres, overcrowding, lack of ventilation and poor prevention practices dramatically increase the risks of TB transmission. Data from sub-Saharan correctional centres indicates an ominous situation. The prevalence of TB in South African correctional centres is estimated to be 6 to 30 times higher than that in the general population, which is a risk for both staff and offenders [23]. Furthermore, prisons in South Africa do not have proper isolation facilities to treat multi- and extensively drug-resistant tuberculosis (MDR-TB and XDR-TB) [23]. Such facilities are also scarce in district and referral hospitals, expounding the problem further. So, the questions remain: where should offenders with XDR-TB be housed and what will happen to them [23]? Overcrowded correctional centres are a breach of United Nations and other international standards, as well as offenders' constitutional rights, which require that all offenders are to be treated with respect to their inherent dignity and value as human beings, including being accorded a reasonable amount of space [24,25]. Furthermore, penile-anal penetration is in general a substantial problem. These findings mirror the UNODC/UNAIDS/World Bank [9] report which reveals that much of the sex among men in offenders is consensual; rape and sexual abuse are often used to exercise dominance in the culture of violence that is typical of correctional centre life. Offender male rape is considered one of the most ignored crimes, and victims of rape and other forms of sexual violence are at higher risk of contracting HIV [8].

### Management and staff capacity

The functioning of the central management in the centre needs to be improved. Increasing staff capacity is clearly a prerequisite for increasing security and safety for staff and offenders. The minimum requirement should be to live up to the existing South African standards that specify the number of staff (for example, security, social workers, teachers and psychologists) per ratio of offenders. For the purposes of this study, of particular importance are the issues mentioned with regard to human resource management. The most urgent problem is understaffing. According to Walmsley [26], when there is growth in correctional centre numbers, the ratio of staff to offenders invariably falls. Reduced staff-to-offender ratios are likely to mean less effective supervision by the staff and less time for them to organise activities to ensure the existence of a positive regime that maximises the chances of former offenders being successfully reintegrated into the community. In particular, treatment programmes, including pre-release courses, are likely to be negatively affected. Furthermore, there are likely to be harmful effects on staff in terms of increased stress and sickness. The World Health Organization [27] substantiates these findings, stating that many correctional centres experience an increasing absence rate due to illness among staff members. Such problems should be addressed by counselling and the supervision of individual staff members. Intervision (group supervision) is one option which has proved to be very effective (and cost effective) for this purpose in other projects conducted by the Trimbos Institute.

### Programmes/activities for offenders

The available range of programmes and activities for offenders is limited, similarly to the study by Taxman et al [28], in which agencies reported a high frequency of providing drug abuse services; the prevalence rates were misleading because less than a quarter of the offenders in correctional centres and less than 10% of those in community correctional agencies had daily access to these services through correctional agencies. In addition, these are predominantly drug treatment services that offered few clinical services [28]. Given that drug-involved offenders are likely to have addiction rates that are four times greater than those among the general public, the drug treatment services and correctional programmes available to offenders do not appear to be appropriate for the needs of this population [28,29]. A needs assessment might be a useful tool to develop programmes and activities which are relevant for and appreciated by offenders. The re-introduction of life-skill programmes should also be considered as they have proved to be effective for the social rehabilitation of offenders after release [28,29]. A closer cooperation between the correctional centre and the community can be helpful for improving the service offered and for assuring the continuity of care, as corroborated in other studies [30].

### Limitations

The findings of this study are subject to the limitations of the study design and sample size. In particular, this study focused on incarcerated youth at one correctional centres, and thus, the findings might not be generalisable to other correctional centres. The sample size of both KI offenders and staff was also small, and thus cannot be regarded as representative of the broader correctional service population. Also, participant inclusion criteria required staff to be committed to improving health situations in the centre; thus staff working at the centre who are not committed to improving the situation may have imparted different views. Furthermore, offender participants could have also had different drug-use knowledge and perceptions to other people and due to the fact that they were incarcerated, may also have held specific views. The analysis of material from the focus groups (as opposed to the individual questionnaires) is also potentially subject to more interviewer and author bias. Although it is obviously important to bear in mind any potential sample representivity shortcoming of biases, ultimately, the aim of the study was not to provide a basis for substantial generalisation, but rather to provide a descriptive account that can inform practices in a practical manner.

## Conclusion

The detection of serious communicable diseases such as HIV infection and TB, accompanied by adequate treatment and the introduction of harm-reduction measures as necessary, can contribute significantly to the health status of the communities from which the offenders come and to which they return. Public health in South Africa cannot afford to ignore correctional centre health. The rise and rapid spread of HIV infection and AIDS, the resurgence of other serious communicable diseases such as TB and the increasing recognition that correctional centres are inappropriate receptacles for people with addiction and mental health problems have thrust correctional centre health high on the public health agenda. In addition, it is now known that drug addiction and mental health conditions can satisfactorily be treated in correctional centres. Outcomes indicate that there are many health problems (inclusive of drug-related health problems), staffing and management aspects. Drug use-related health risks are largely related to transactions and power imbalances linked to drug supply, control and demand. HIV risk seems to be related primarily to penal-anal intercourse. There is also a possibility that IDU in such settings can exacerbate HIV infection risks. Furthermore, offenders do not currently have access to appropriate activities while in correctional services. Study findings suggest that the time in custody should be used to promote healthier lifestyles. Furthermore, national strategies need to include Correctional Service policies, as correctional centres contain, at any one time, a disproportionate number of those requiring health assistance. In South Africa, the national voluntary HIV counselling, testing and treatment campaign (HCT) should support expanded HIV/TB testing and treatment in correctional centres. Good correctional centre health prevents the spread of diseases and promotes health through awareness of what everyone can do to help maintain their own health and well-being and that of others. In addition, it can help improve the health status of communities, thus contributing to health for all.

## Competing interests

An abstract poster for this article was presented at XIX International AIDS Conference, 22–27 July 2012, Washington DC, United States of America, and an unpublished report related to the study has been drafted. The authors declare that they have no competing interests.

## Authors’ contributions

MMLDS drafted the original article manuscript, adapted the questionnaire, assisted with focus groups interviews, managed the data collection and analysis together with the fieldworkers and contributed to the development of policy recommendations. FT advised and assisted with the focus group interviews, interpretation of the data, technical quality of the paper and the development of policy recommendations based on the outcomes of the study. GW sourced the funding and partnerships for this study and also contributed to the technical quality of this paper. RP assisted with the questionnaire design, individual interviews and technical quality of this paper. All authors read and approved the final manuscript.
